# The Odontological Society

**Published:** 1896-06

**Authors:** 


### The Odontological Society.

The Odontological Society of Cincinnati held its monthly meeting March 27th at the office of Dr. Vandervort. A fair representation of the membership was present. A paper on "Hypnotic Suggestion" was read by Dr. A. A. Kumler which contained some interesting points that drew out considerable discussion on the question, The generally-expressed opinion of the members was to the effect, that hypnotism, while it may be employed in some cases with benefit, its general use in medical or dental practice does not seem feasible. In some cases it can be used effectively upon patients, but again there is such a great want of accurate and definite knowledge upon the subject as to make its use impracticable; and, furthermore, there is almost a universal prejudice against its use, and there are very few persons who wish to array themselves squarely against so universal a prejudice. It was conceded on all sides that there was much in hypnotism, and that perhaps further knowledge would render it more useful and practicable than it has been up to the present time. It is possible, and even probable, that investigation will in the future clear away some, or even many, of the difficulties that have hitherto attended this subject.

